# EphA1 activation promotes the homing of endothelial progenitor cells to hepatocellular carcinoma for tumor neovascularization through the SDF-1/CXCR4 signaling pathway

**DOI:** 10.1186/s13046-016-0339-6

**Published:** 2016-04-11

**Authors:** Yi Wang, Haitao Yu, Yunfeng Shan, Chonglin Tao, Fang Wu, Zhengping Yu, Pengyi Guo, Jianfei Huang, Junjian Li, Qiandong Zhu, Fuxiang Yu, Qitong Song, Hongqi Shi, Mengtao Zhou, Gang Chen

**Affiliations:** Environmental and Public Health School of Wenzhou Medical University, Wenzhou, 325000 China; Department of Hepatobiliary Surgery, The First Affiliated Hospital, Wenzhou Medical University, Wenzhou, 325000 China; Department of Gastroenterology, The First Affiliated Hospital, Wenzhou Medical University, Wenzhou, 325000 China

**Keywords:** EphA1, SDF-1/CXCR4, Endothelial progenitor cells, Hepatocellular carcinoma, Neovascularization

## Abstract

**Background:**

Endothelial progenitor cells (EPCs) can migrate to the tumor tissue and enhance the angiogenesis of hepatocellular carcinoma (HCC); thus, they are associated with a poor prognosis. However, the specific molecular mechanism underlying the homing of EPCs to the HCC neovasculature remains unrevealed.

**Methods:**

Co-culture experiments of endothelial progenitor cells with HCC cells with modulation of EphA1 were performed in vitro. Using EPCs as angiogenic promoters by injecting them into HCC xenograft-bearing nude mice via their tail veins to test homing ability of EPCs changed according to different EphA1 level in HCC xenograft.

**Results:**

In this study, we found that the up-regulation of EphA1 expression in HCC cells could affect not only the chemotaxis of EPCs to tumor cells and endothelial cells (ECs) but also the tube formation ability of EPCs in a paracrine fashion. Further, we revealed that the increased expression of EphA1 in HCC cells led to an increased SDF-1 concentration in the tumor microenvironment, which in turn activated the SDF-1/CXCR4 axis and enhanced the recruitment of EPCs to HCC. In addition, the EphA1-activated SDF-1 expression and secretion was partially mediated by the PI3K and mTOR pathways. In vivo experiments demonstrated that blocking EphA1/SDF-1/CXCR4 signaling significantly inhibited the growth of HCC xenografts. Using immunohistochemistry and immunofluorescence assays, we verified that the inhibition of tumor angiogenesis was at least partially caused by the decreased number of EPCs homing to tumor tissue.

**Conclusions:**

Our findings indicate that targeting the EphA1/SDF-1 signaling pathway might be a therapeutic anti-angiogenesis approach for treating HCC.

**Electronic supplementary material:**

The online version of this article (doi:10.1186/s13046-016-0339-6) contains supplementary material, which is available to authorized users.

## Background

Hepatocellular carcinoma (HCC) is the most common primary liver tumor; it is the second leading cause of cancer-related death worldwide, and its incidence is increasing yearly [1]. HCC is often diagnosed at late stages when only limited therapeutic options are available; consequently, the survival rate for HCC patients is dismal. Thus, there is an urgent need to develop novel therapeutic approaches for advanced HCC. Research has confirmed that angiogenesis is required for invasive tumor growth and metastasis; thus, angiogenesis is an important point in controlling the malignant progression of tumors, and angiogenesis inhibition may be a valuable new approach for cancer therapy. For example, drugs targeting angiogenic pathways, such as sorafenib, have been proven to have specific antitumor effects [2]. Clinically, however, sorafenib treatment has produced only modest survival benefits in most patients and is not considered curative [3]. Therefore, the development of anti-angiogenic strategies that are more effective for treating HCC remains a challenging task.

A number of studies suggest that the recruitment of circulating endothelial progenitor cells (cEPCs) contributes greatly to tumor neovascularization and that tumor growth could be inhibited by retarding the incorporation ability of EPCs [4–7]. Researchers have found that EPCs can serve as a critical regulator of tumor progression from micrometastases to macrometastases [8]. Using an orthotopic liver tumor model, Li et al. confirmed that the inhibition of the EPC population could suppress liver tumor metastasis [9]. In addition, our previous study verified that bone marrow-derived EPCs can home to the tumor tissue of orthotopic HCC xenografts [10]. Nevertheless, despite the strong evidence supporting the promotive role of these cells on neovascularization in HCC, the mechanisms underlying EPCs’ homing and recruitment to neovessels in HCC remain unclear.

The erythropoietin-producing human hepatocellular carcinoma (Eph) receptors, which comprise the largest family of receptor tyrosine kinases (RTKs), are involved in normal tissue remodeling and cancer progression, especially in tumor angiogenesis [11]. Ephrins/Ephs engage in critical steps of angiogenesis, including juxtacrine cell–cell contacts, cell adhesion to extracellular matrix, and cell migration [12]. Recent evidence also suggests the important role of ephrins/Ephs in regulating EPCs’ recruitment and homing [13, 14]. EphA1, the first identified Eph family member, is physiologically expressed in epithelial tissues, and its pathological overexpression has been reported in a variety of tumors, including gastric cancer [15], liver cancer [16], prostate cancer [17], and esophageal squamous cell carcinoma [18]. In a previous study, an artificial overexpression of EphA1 in fibroblasts was found to be tumorigenic in nude mice [19]. Iida et al. reported that EphA1 and its ligand ephrinA1 were overexpressed in the tumor cells of HCC and that exogenous ephrinA1-Fc fusion could lead to increased EphA1 expression and activation of its downstream signaling [16]. Our previous study also found that the targeted suppression of EphA1 expression significantly inhibited the tumor growth of HCC xenografts, mainly by suppressing tumor angiogenesis [20]. Although research suggests a close link between EphA1 and HCC angiogenesis, the question of whether the homing of EPCs to HCC neovasculature is regulated by ephrinA1 and its receptor EphA1 remains unanswered.

In this study, we used EPCs as angiogenic promoters by injecting them into HCC xenograft-bearing nude mice via their tail veins to favor tumor development. By manipulating the expression of EphA1 in xenografted tumor tissue and in cultured HCC cells, we observed that the up-regulation of EphA1 expression in HCC cells promotes the chemotaxis of EPCs to tumors and endothelial cells through a paracrine mechanism. Further, both in vivo and in vitro experiments confirmed the pivotal role of the SDF-1/CXCR4 axis in mediating the promotive effect of EphA1 on EPCs’ homing to tumor neovasculature. Our findings indicate that targeting the EphA1/SDF-1 signaling pathway might provide a therapeutic anti-angiogenesis approach to HCC.

## Results

### Phenotypic and functional characterization of EPCs derived from peripheral blood

We successfully isolated EPCs from adult peripheral blood samples (n = 13) and expanded in vitro. EPC colonies were verified on the 7th day (Additional file 1: Figure S1a) and were expanded through another 2 weeks of culture. The cells exhibited a cobblestone-like morphology typical of ECs (Additional file 1: Figure S1a). Moreover, the cell phenotype was identified using IF assay. The EPCs were uniformly positive for several endothelial markers, including CD31, CD133, eNOS, and VEGFR2 (Additional file 1: Figure S1b), and they were able to incorporate acetylated-LDL and bind the lectin UEA-1 (Additional file 1: Figure S1c). Importantly, the EPCs were found to lack the expression of the hematopoietic antigens CD45 and CD90 (Additional file 1: Figure S1b). Furthermore, Matrigel assays revealed that the EPCs were characterized by their ability to form a network of tube-like structures (Additional file 1: Figure S1d). These results are consistent with previous reports [21, 22].

### EphA1 regulates EPCs’ chemotaxis and tube formation potency

**Fig. 1:**
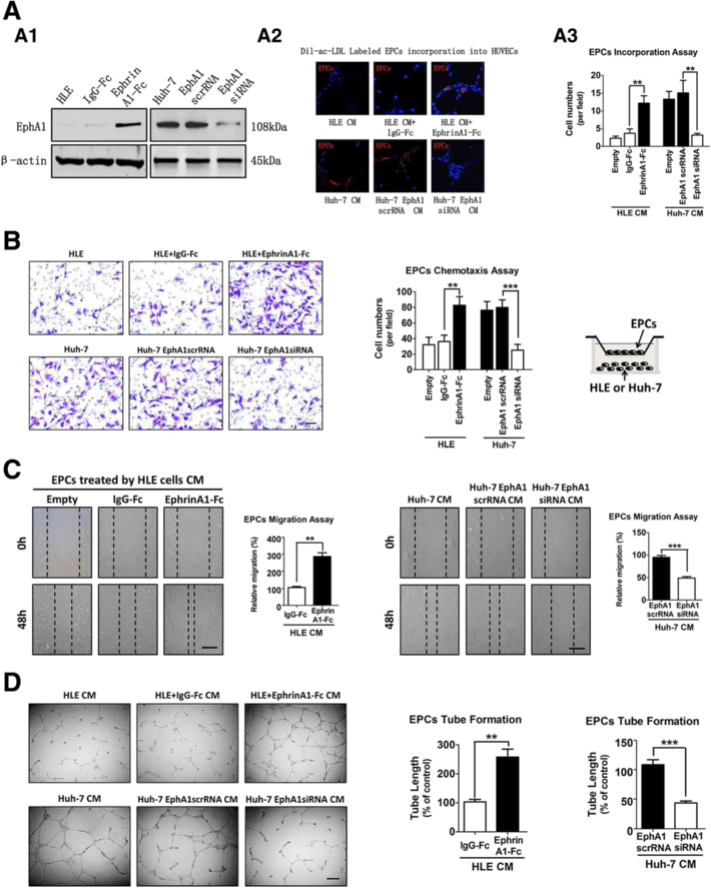
EphA1 regulates EPC proangiogenic potency in a paracrine fashion in vitro. **a** A1: WB assay showing the EphA1 protein expression in HCC cells. A2: EPC incorporation into HUVECs, EPC’s uptake of DiI-ac-LDL (*red*) together with HUVEC tube formation (*blue*). A3: EPC incorporation assay analysis data (**P < 0.01). The data are expressed as the means ± SD of three independent experiments. **b** Boyden chamber staining of EPCs’ chemotaxis to HCC cells with different EphA1 levels, *Asterisks* indicate significant differences (**P < 0.01, ***P < 0.001) with respect to the corresponding control. The data are expressed as the mean ± SD of three independent experiments. **c**: EPC migration. **d** EPC tube formation. The migration and tube formation of EPCs were measured after treatment with conditioned medium from HCC cells with different EphA1 levels. The data are expressed as the mean ± SD of three independent experiments. *Asterisks* indicate significant differences (**P < 0.01, ***P < 0.001) with respect to the corresponding control

EphrinA1 has two native receptors, EphA1 and EphA2. EphA1 is overexpressed in the tumor tissue in HCC patients [16, 23]. Therefore, we investigated the role of EphA1 in EPCs’ chemotaxis and tube formation potency in HCC cells through manipulating EphA1 gene expression in HLE cells (EphA1 low expression hepatoma cells) and Huh-7 cells (EphA1 high expression hepatoma cells). EphrinA1-Fc was used to increase EphA1 expression in HLE cells. The western blot (WB) assay showed that ephrinA1-Fc recombination protein increased the expression of EphA1 but not EphA2 (Additional file 2: Figure S2a), consistent with the results of immunofluorescence staining (Additional file 2: Figure S2b). The result not only confirmed the success of the EphA1 expression manipulation, but also ruled out the possibility that EphA2 is involved as a receptor in ephrinA1-Fc-activated downstream signaling in HLE cells, despite a previous finding that ephrinA1-Fc can up-regulated EphA2 expression and activate its downstream signaling in endothelial cells [24]. Further, the WB assay verified that EphA1 siRNA successfully suppressed EphA1 expression in Huh-7 cells (Fig. 1a1). Subsequently, a Transwell assay was conducted to investigate the effect of EphA1 upregulation on EPC chemotaxis to HCC cells. The results demonstrated that the enhanced EphA1 expression in EphA1-negative HLE cells resulting from ephrinA1-Fc recombinant protein increased the chemotaxis of EPCs to HLE cells, while the decreased EphA1 expression resulting from EphA1 siRNA transfection in EphA1-positive Huh7 cells had the opposite effect (Fig. 1b). Next, EPC incorporation into the tubular networks formed by HUVEC was evaluated. The EPC incorporation assay showed that the number of Dil-ac-LDL labeled EPCs incorporation into HUVECs increased after treatment with conditional medium (CM) from the EphA1 high expression HCC cells (HLE cells treated with ephrinA1-Fc recombinant protein or Huh-7 cells transfected with EphA1 scrRNA) compared to treatment with CM from the EphA1 low expression HCC cells (HLE cells treated with IgG-Fc recombinant protein or Huh-7 cells transfected with EphA1 siRNA) (Fig. 1a2, a3). Furthermore, wound healing and tube formation assays were conducted to investigate whether EphA1 up-regulation in HCC cells promotes EPCs’ potency for physiological angiogenesis. The results demonstrated that the migration ability of EPCs remarkably increased after treatment with conditioned medium from EphA1-negative HLE cells stimulated with recombinant human ephrinA1-Fc and decreased after treatment with conditioned medium from EphA1 siRNA-transfected EphA1-positive Huh7 cells (Fig. 1c). The same tendency was found in the tube formation assay (Fig. 1d). These results indicate that the increased expression of EphA1 in HCC cells promotes the chemotactic and tube formation abilities of EPCs in the tumor microenvironment.

### EphA1 activates SDF-1/CXCR4 signaling in HCC

**Fig. 2:**
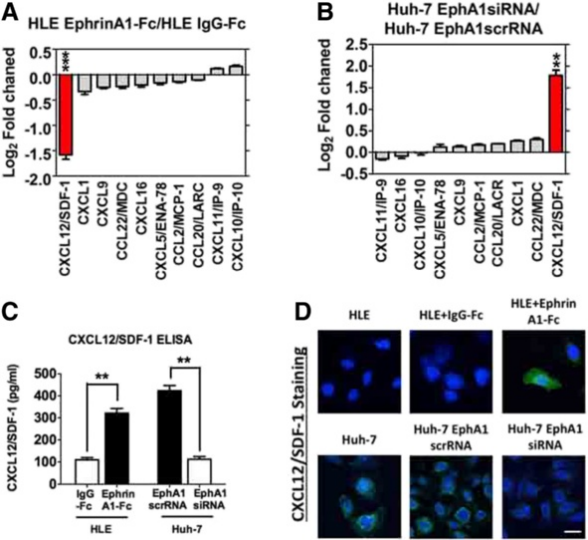
Up-regulated EphA1 expression in HCC cells promotes SDF-1 expression and secretion. A-B. Chemokine mRNA expression profile determined with qRT-PCR. The data represent the mean ± SD of three independent experiments. **a** HLE cells: The result of ephrinA1-Fc-activated HLE cells is normalized to the expression in the IgG-Fc-activated HLE cells for each chemokine. The red bar represents the relative SDF-1 mRNA expression level in the ephrinA1-Fc-activated HLE cells. *Asterisks* indicate significant differences (***P < 0.001) with respect to the IgG-Fc-activated HLE cells. B. Huh-7 cells with different EphA1 expression levels: The result is normalized to the expression in Huh-7 cells transfected with EphA1 scrRNA for each chemokine. The red bar represents the relative SDF-1 mRNA expression level in Huh-7 cells transfected with EphA1 siRNA. *Asterisks* indicate significant differences (**P < 0.01) with respect to the Huh-7 cells transfected with EphA1 scrRNA. **b** SDF-1 protein concentration, evaluated with ELISA, in conditioned medium from HCC cells with different EphA1 levels. The data represent the mean ± SD of three independent experiments. *Asterisks* indicate significant differences (**P < 0.01). **c** IF staining of SDF-1 (*green*) and DAPI (*blue*) in HCC cells (top panel: HLE cells; bottom panel: Huh-7 cells). Images were obtained using a confocal laser scanning microscope. Magnification: × 400; scale bar, 20 μm

Chemokines have been verified as the key factors mediating EPCs’ homing for tumor neovascularization [25]. To verify whether EphA1 can increase chemokine expression and secretion in HCC cells, we detected 10 chemokines that are known to be associated with HCC [26–35] and designed specific primers for PCR assays (Table 1). Using qPCR and ELISA, we found that among all of the detected chemokines, the up-regulation of EphA1 expression in ephrinA1-Fc-stimulated HLE cells resulted in the most significant increase in SDF-1 mRNA expression in cells (Fig. 2a) and protein secretion into the culture medium (Fig. 2c). Consistently, the inhibition of EphA1 expression by EphA1 siRNA in EphA1-positive Huh7 cells led to decreased mRNA expression (Fig. 2b) and protein secretion (Fig. 2c) in SDF-1. The findings from immunofluorescence staining with SDF-1 (green) and DAPI (blue) were consistent with those of the qPCR assay (Fig. 2d).

**Table 1:** The primer sequences of Chemokines used in this article

Target gene	Forward sequence	Reverse sequence
CXCL1	CATCGAAAAGATGCTGAACAGT	ATAAGGGCAGGGCCTCCT
CXCL5	CCTTTTCTAAAGAAAGTCATCCAGA	TGGGTTCAGAGACCTCCAGA
CXCL9	CTGGAGCAGTGTGGAGTTC	CCGTTCTTCAGTGTAGCAATG
CXCL10	GAACTGTACGCTGTACCTGCA	TTGATGGCCTTCGATTCTGGA
CXCL11	CAGAGAGGCTGAGACCAACC	GCTGAAGGTGTGAGCTTTGG
CXCL12	CTCGGGATGTGTAATGGCT	GCCTCCATGGCATACATAGA
CXCLl16	ACTCGTCCCAATGAAACCAC	ATGAAGATGATGGCCAGGAG
CCL2/MCP-1	GCATCCACGTGTTGGCTCA	CTCCAGCCTACTCATTGGGATCA
CCL20	GCCTCTCGTACATACAGACGC	CCAGTTCTGCTTTGGATCAGC
CCL22/MDC	TGCGCGTGGTGAAACACTT	TAGGCTCTTCATTGGCTCAGCT

**Fig. 3:**
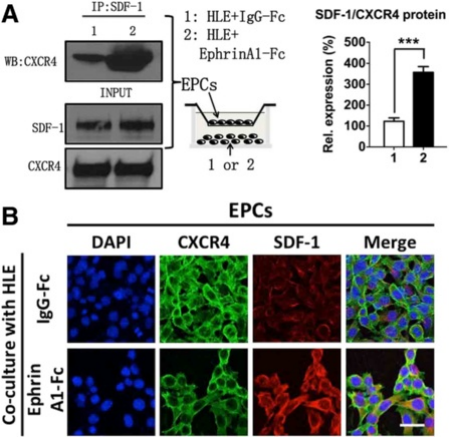
EphA1 increases the binding of SDF-1 to CXCR4 in EPCs. **a** Cell lysates from EPCs co-cultured with IgG-Fc-activated (Lane 1) or ephrinA1-Fc-activated HLE cells (Lane 2) were used for co-IP using anti-SDF-1 antibodies. WB was performed using antibodies against CXCR4, with β-actin as a control for protein loading. The histogram on the right shows the semi-quantification results of WB. The data represent the mean ± SD of three independent experiments. The results show that the activation of EphA1 leads to an increased pull-down of CXCR4 in EPCs. **b** IF staining of EPCs after co-culturing with IgG-Fc-activated (*top panel*) or ephrinA1-Fc-activated HLE cells (*bottom panel*) using anti-CXCR4 antibody (*green fluorescence*), anti-SDF-1 antibody (*red fluorescence*), and DAPI (blue fluorescence). Yellow fluorescence in the merged images indicates the co-expression of SDF-1 and CXCR4 in EPCs after co-culturing with HLE cells activated by IgG-Fc or ephrinA1-Fc. Images were obtained using a confocal laser scanning microscope. Magnification: × 400; scale bar, 10 μm

To further verify whether SDF-1/CXCR4 signaling is the key contributor to EPCs’ homing process in response to the EphA1 level change in HCC cells, we performed an IP assay using the lysates from EPCs after they were co-cultured with IgG-Fc- or ephrinA1-Fc-stimulated HLE cells and the SDF-1 antibody, followed by a WB assay of the immunoprecipitated eluate (IP eluate) for CXCR4. The results demonstrated that the IP eluate from the cells with increased SDF-1 expression had a remarkably higher CXCR4 level, suggesting that SDF-1 and CXCR4 interact with each other (Fig. 3a). In addition, using immunofluorescence (IF), we verified that compared with the control group, EPCs co-cultured with ephrinA1-Fc-activated HLE cells showed significant SDF-1 and CXCR4 co-expression (Fig. 3b).

To rule out the possibility of EphA2 involvement, we successfully constructed EphA2 siRNA to knock down the EphA2 gene, as evidenced by the fact that EphA2 siRNA-transfected Huh-7 cells showed a significantly lower EphA2 mRNA expression level compared with EphA2 scrRNA-transfected Huh-7 cells (Additional file 2: Figure S2c). Subsequently, a qPCR assay revealed that EphA2 siRNA-transfected Huh-7 cells did not exhibit any significant changes in the mRNA expression levels of any of the detected chemokines (Additional file 2: Figure S2d).

The above results indicate that EphA1 is the key receptor responding to ephrinA1-Fc stimulation in HLE cells and that the up-regulation of EphA1 expression can activate SDF-1/CXCR4 signaling in the tumor microenvironment.

### SDF-1 inhibition suppresses the EphA1-induced chemotaxis and tube formation ability of EPCs

**Fig. 4:**
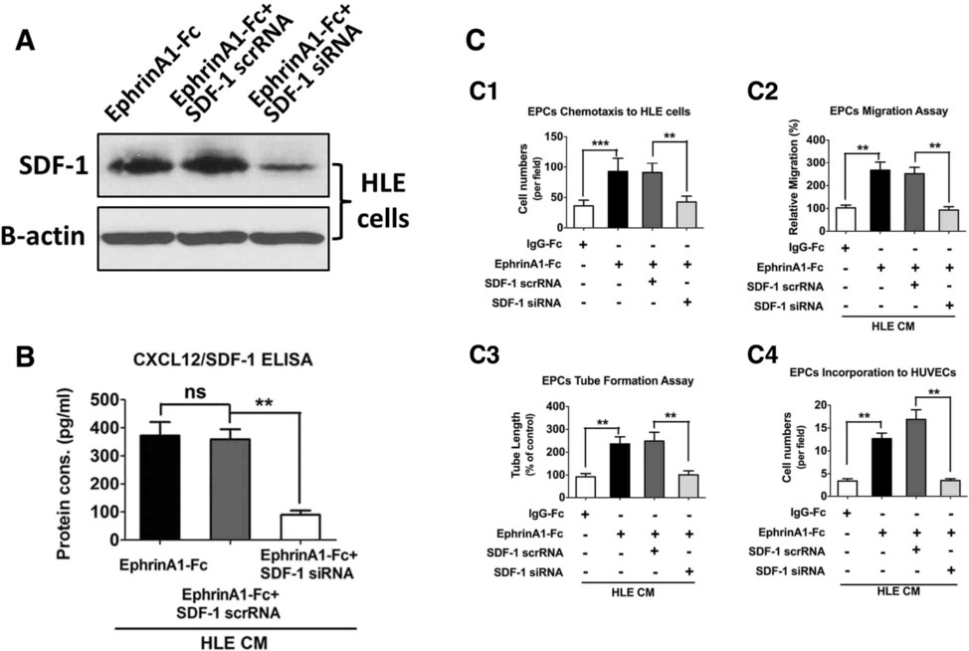
The effect of EphA1 on HCC angiogenesis is SDF-1 dependent in vitro. **a** WB of SDF-1 in ephrinA1-Fc-activated HLE cells transfected with SDF-1 scrRNA or SDF-1 siRNA, with β-actin as a control for protein loading. **b** The SDF-1 protein concentration in conditioned medium from ephrinA1-Fc-activated HLE cells transfected with SDF-1 scrRNA or SDF-1 siRNA, evaluated with ELISA. *Asterisks* indicate significant differences (**P < 0.01), and “ns” indicates an insignificant difference. **c** The abilities of EPCs to incorporate into the HUVECs (C1), migrate (C2), chemotax to HLE cells (C3), and form tubular structures (C4) were measured after treatment with conditioned medium from ephrinA1-Fc-activated HLE cells transfected with SDF-1 scrRNA or SDF-1 siRNA. The data represent the mean ± SD of three independent experiments. *Asterisks* indicate significant differences (**P < 0.01; ***P < 0.001)

To investigate whether SDF-1 inhibition blocks the EphA1-activated chemotaxis and tube formation ability of EPCs, we transfected SDF-1 siRNA into ephrinA1-Fc-stimulated HLE cells with an up-regulated EphA1 expression and used scrambled siRNA as a control. WB showed decreased SDF-1 protein expression and secretion (Fig. 4a, b), confirming the success of the SDF-1 gene knockdown. The Transwell experiment demonstrated that compared with the control scrambled RNA, the SDF-1siRNA knockdown of HLE cells with an activated EphA1 expression led to a significant decrease in EPC chemotaxis (Fig. 4c1). In addition, after treatment with conditioned medium from SDF-1 knockdown ephrinA1-Fc-activated HLE cells, EPCs’ abilities to migrate (Fig. 4c2), form a tubular structure (Fig. 4c3), and incorporate into mature EC populations (Fig. 4c4) were all suppressed compared with the SDF-1 scrambled RNA group. These results suggest that targeting SDF-1 might decrease the EphA1-induced chemotaxis and tube formation ability of EPCs in the tumor microenvironment.

### EphA1 promotes SDF-1 expression in HCC cells through the Akt and mTOR pathway

**Fig. 5:**
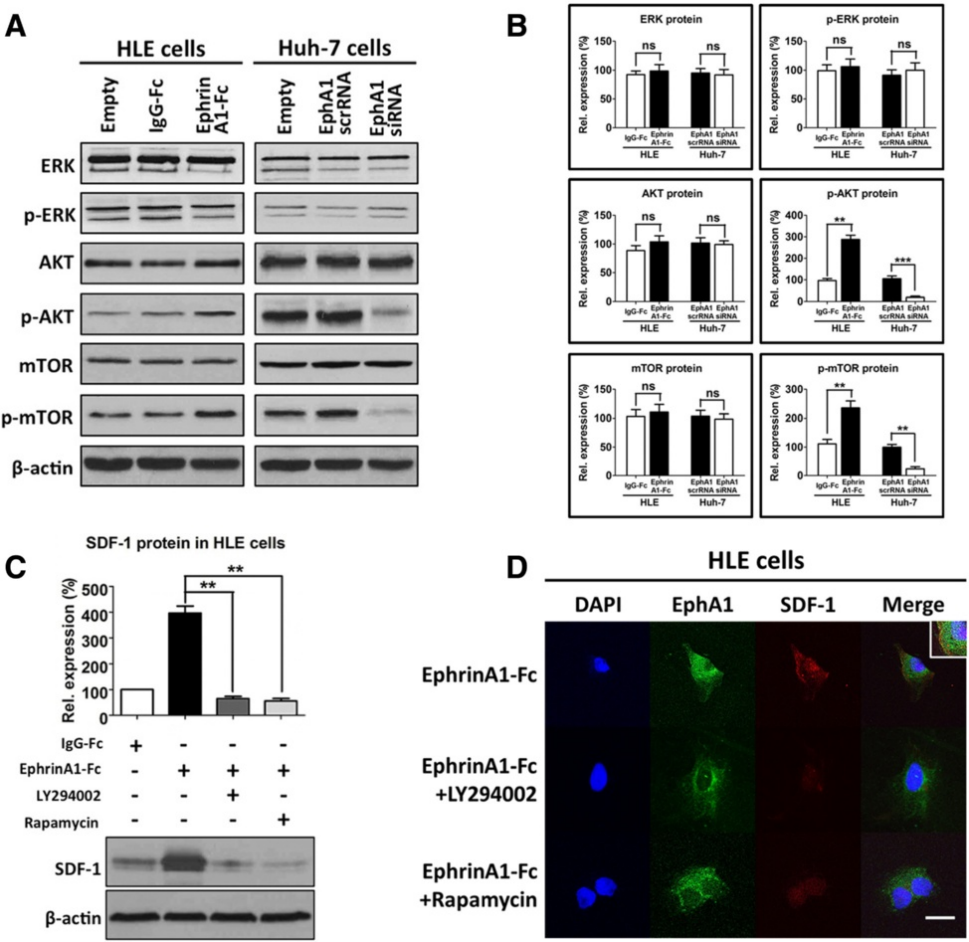
EphA1 increases SDF-1 expression via activation of the Akt and mTOR pathways. **a** WB assay of the EphA1 downstream signaling molecules ERK, AKT and mTOR, expressed in untreated and treated HLE cells as well as Huh-7 cells with manipulated EphA1 levels, with β-actin as a control or protein loading. **b** Histograms show the relative (Rel) protein expression results from WB, which were normalized to the expression level in untreated cells. The data represent the mean ± SD of three independent experiments. *Asterisks* indicate significant differences (**P < 0.01; ***P < 0.001), and “ns” indicates an insignificant difference. **c** The WB assay of SDF-1 in IgG-Fc- or ephrinA1-Fc-activated HLE cells after the respective blockage of the Akt and mTOR pathways with the corresponding specific inhibitors LY294002 (2 ng/ml) and rapamycin (2 ng/ml). Β-actin served as a control for protein loading. The data represent the mean ± SD of three independent experiments. *Asterisks* indicate significant differences (**P < 0.01) **d** Double IF staining of SDF-1 (*green*) and EphA1 (*blue*). Images were obtained using a confocal laser scanning microscope. The results show that SDF-1 expression decreases after the blockage of the Akt and mTOR pathways with their respective specific inhibitors, LY294002 (2 ng/ml) and rapamycin (2 ng/ml). Magnification: × 400; scale bar: 10 μm

To identify the pathway involved in the EphA1-activated expression and secretion of SDF-1 in HCC cells, we first examined the protein expression of three EphA1 downstream signaling molecules, ERK, Akt and mTOR. WB results revealed that the active EphA1 in ephrinA1-Fc-stimulated HLE cells increased the protein expressions of phosphor-Akt and phosphor-mTOR; however, no difference in the protein level of phosphor-ERK was observed. Consistently, the knockdown of EphA1 expression by EphA1 siRNA in EphA1-positive Huh7 cells exhibited the opposite changes (Fig. 5a, b). Next, using LY294002 (an inhibitor of the Akt signaling pathway) and rapamycin (an inhibitor of the mTOR signaling pathway), we found that blocking the Akt and mTOR pathway in HCC cells decreased EphA1-activated SDF-1 expression (Fig. 5c). The findings from immunofluorescence staining with EphA1 (green), SDF-1 (red), and DAPI (blue) were consistent with those of the WB assay (Fig. 5d). These results suggest that the Akt and mTOR pathway mediates the enhancement of SDF-1 expression and secretion by EphA1 in HCC cells.

### EphA1 promotes EPCs’ homing for neovascularization of HCC xenografts

**Fig. 6:**
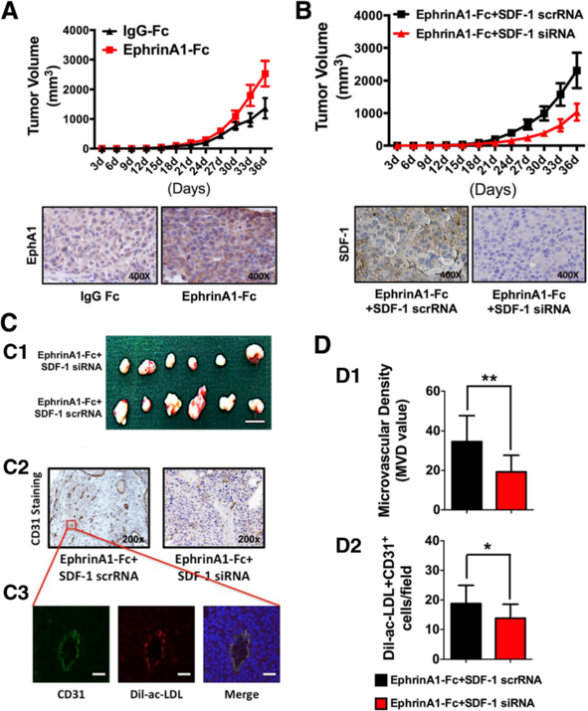
Targeted SDF-1 inhibition suppresses EphA1-mediated HCC angiogenesis and EPCs’ homing process in vivo. HLE cells were injected in 6-week-old nude nu/nu (CD-1) mice to develop tumors. When a tumor mass was evident (14 days), Dil-ac-LDL-labeled EPCs were intravenously injected into the mice through the tail vein, as described in the Methods section. **a** Comparison of tumor growth between the IgG-Fc groups and ephrinA1-Fc groups (*top panel*) and the EphA1 expression levels in these two groups (*bottom panel*). **b** Comparison of tumor growth between the ephrinA1-Fc + SDF-1 scrRNA group and ephrinA1-Fc + SDF-1 siRNA group (*top panel*) the SDF-1 expression in these two groups (*bottom panel*): The tumor growth curves were obtained by measuring the tumor volume every 3 days. **c** Top: Representative photograph of the xenograft of HLE cells treated with ephrinA1-Fc to activate EphA1 expression and transfected with SDF-1 siRNA (*top panel*) or scrRNA (*bottom panel*); middle: IHC staining images of tumors from each group using anti-CD31 antibody; bottom: high-magnification cross-section images of one single vessel selected from the IHC image of the ephrinA1-Fc + SDF-1 siRNA group (indicated by the red box in the image in the middle panel). As the images indicate, EPCs labeled 1DiI (*red*) also express the EC marker CD31 (*green*) and are mainly located in the tumor vasculature (×200; scale bar, 50 μm). **d** Top: Microvascular density of HLE cells with ephrinA1-Fc-activated EphA1 expression transfected with SDF-1 siRNA (*red bar*) or scrRNA (*black bar*) in the xenografts; Bottom: Number of EPCs homing to the tumor vasculature of HLE cells with ephrinA1-Fc-activated EphA1 expression transfected with SDF-1 siRNA (*red bar*) or scrRNA (*black bar*) in the xenografts

**Fig. 7:**
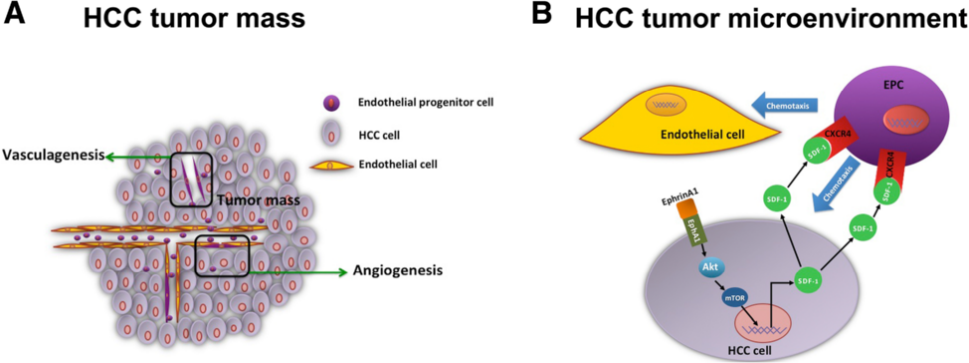
Working model of how the EphA1-activated SDF-1/CXCR4 signaling promotes EPCs’ homing to HCC neovascularization. **a** In the HCC tumor mass, cEPCs incorporate into the EC layers and tumor vessels (angiogenesis) or migrate to non-vascularized tumor sites to facilitate the initial establishment of the tumorous endothelium, contributing to angiogenesis by providing structural support for nascent vessels (vasculogenesis). **b** In the tumor microenvironment, ephrinA1/Fc-forced expression of EphA1 activates its downstream signaling molecular AKT and mTOR and then increases the expression and secretion of SDF-1 to the tumor microenvironment. Secreted SDF-1 in turn binds with its receptor CXCR4 on the EPC cell membrane and contributes to the increased angiogenic potency of EPCs and their ability to chemotax to ECs and tumor cells

To further clarify whether targeting SDF-1 can suppress EphA1-induced HCC growth and EPCs’ homing process, we established HLE cell xenograft models with different EphA1 levels in mice, which were then injected with EPCs through the tail vein on the post-xenograft 14th day. After 36 days, the tumors containing HLE cells with activated EphA1 had a significantly greater volume than the tumors containing HLE cells stimulated with control IgG-Fc (Fig. 6a). In addition, compared with SDF-1 scrambled RNA, the SDF-1 siRNA-treated HLE cells with activated EphA1 expression showed remarkably smaller resulting tumors (Fig. 6b, c1), suggesting a pivotal role of SDF-1 in EphA1-enhanced tumor growth. Next, we investigated the effect of targeting SDF-1 on EphA1-induced HCC angiogenesis by comparing the microvascular density (MVD) of the xenografts of mice injected with SDF-1 siRNA-treated HLE cells and those treated with SDF-1 scrambled RNA-treated HLE cells. MVD was determined based on the IHC staining of tumor tissue for CD31. The results indicate that compared with the scrambled RNA control group, SDF-1 knockdown by siRNA significantly inhibited MVD in the tumors containing HLE cells with activated EphA1 expression (Fig. 6c2, d1). Next, we further verified the role of SDF-1 in the EphA1-promoted recruitment of EPCs to HCC for vascularization as a key downstream molecule. Dil-ac-LDL labeling was used to track the homing of EPCs to the tumors’ CD31-positive vasculature (Fig. 6c3), and the number of EPCs per microscope field of tumor tissue was counted. The results revealed that compared with the SDF-1 scrambled RNA-transfected cells with activated EphA1 expression, the HCC xenografts derived from SDF-1 siRNA-knockdown HLE cells had a smaller number of EPC cells homing to the tumor vasculature (Fig. 6d2). These results indicate that the activation of EphA1 expression promoted angiogenesis in HCC and that targeting SDF-1 might be an effective tool for inhibiting EphA1-induced HCC angiogenesis and EPC recruitment. The working model of how the EphA1-activated SDF-1/CXCR4 signaling promote EPCs’ homing to HCC neovascularization was presented (Fig. 7).

## Discussion

By constructing an orthotopic tumor in rat livers and injecting EPCs through the tail vein after a xenograft tumor developed, we preliminarily confirmed that EPCs can specifically chemotax to HCC tumor tissue [20]. However, the definitive molecular mechanism underlying this homing process remains to be clarified. In the present study, we observed that up-regulated EphA1 expression in HCC cells increased SDF-1 expression and secretion in the tumor microenvironment, which in turn not only promoted the chemotaxis, tube formation and migration ability of EPCs but also enhanced tumor growth and microvascular density. Importantly, in vivo experiments revealed that an increased number of EPCs homed to neovessels in HCC. Moreover, this study found that EphA1-activated SDF-1 expression and secretion was mediated by Akt and mTOR pathway.

Previous studies have reported an elevated expression level of EphA1 and its analogue, EphA2, in the tumor tissue of HCC patients [16, 36]. Our previous study also showed that the targeted inhibition of EphA1 could inhibit tumor growth and angiogenesis in HCC [20]. In this study, we used ephrinA1 fusion protein and verified that ephrinA1 can activate EphA1 but not EphA2 receptors in an EphA1-negative HCC cell line of HLE cells (Additional file 2: Figure S2), which was similar to Iida’s findings [16].

Tumor vasculatures develop mainly through angiogenesis via endothelial sprouting from the existing vessels, and this sprouting process is based on the recruitment, proliferation and tube formation of EPCs [37, 38]. The homing of cEPCs to the tumor participates in the angiogenesis process by differentiating and incorporating into the EC populations of the neovessels of HCC or into the tumors themselves. A number of studies have revealed that ephrin/Eph family members can regulate the recruitment and homing of EPCs to tumors. For example, Foubert et al. found that the inhibition of EphB4 activity in EPCs significantly reduced EPC-induced angiogenesis [13] and further revealed that EPCs pretreated with ephrin-B2 have a proangiogenic potential for therapeutic neovascularization [23]. Similarly, Salvucci et al. [39] reported that the EphB2/ephrinB2 signaling pathway could promote EPC chemotaxis and accelerate vascular branching and remodeling. The ephrinA1/EphA1 pathway was also found to be involved in the malignant biological behavior of HCC tumor cells [16]. However, the relationship between the ephrinA1/EphA1 pathway and EPC homing ability in HCC remains to be determined. In this study, the Boyden chamber assays showed that the chemotaxis of EPCs to the ECs and tumor cells was significantly enhanced in the conditioned medium from HLE cell cultures with activated EphA1. The results of these and other assays, allowed us to show for the first time that the ephrinA1/EphA1 pathway activated in HCC can promote EPCs homing process in a paracrine fashion.

Recent studies have found that in addition to the EPCs’ physical contribution to newly formed vessels, the enhanced secretion of chemokines might be a supportive mechanism underlying the homing process of EPCs to tumor neovasculature [25]. As one of the most important chemokines involved in the angiogenesis of HCC [30], SDF-1 mediates the homing of cEPCs to neovessels by binding to the receptor CXCR4 on EPCs [40]. In this study, the activation of the ephrinA1/EphA1 signaling pathway in HCC cells led to an elevated SDF-1 protein level in the HCC tumor microenvironment. Moreover, targeting SDF-1 with siRNA suppressed the ephrinA1/EphA1-enhanced potential of EPCs to migrate to the tumor cells. In in vivo experiments, SDF-1 blockage reversed the promotive effect of ephrinA1-Fc against tumor growth and angiogenesis in the nude mouse HCC xenograft model. Meanwhile, the number of EPCs in the tumor neovessels drastically decreased. Therefore, we believe that the reduction of EPC recruitment might be a major contributor to the change in tumor angiogenesis resulting from SDF-1/CXCR4 axis blockage. In HCC, previous studies have verified that SDF-1/CXCR4 axis could be up-regulated by TIMP-1 [41] and urokinase [42]. Interestingly, tacrolimus, an immunosuppressor, can also promote HCC by activating SDF-1/CXCR4 axis [43]. Here, for the first time, we observed that the SDF-1/CXCR4 axis can be activated by the increased expression of EphA1 in HCC cells in the tumor microenvironment. This finding is similar to a previous report that EphB2 and EphB4 might possess the ability to up-regulate the expression of SDF-1 [39]. In addition to SDF-1, other chemokines, such as CXCL16/CXCR6 [44], CXCL10/CXCR3 [28], and CCL5/CCR5 [45], might play a regulatory role in EPCs mobilization and recruitment. However, we did not notice any significant gene expression changes in these chemokines in HCC cells in response to EphA1 activation.

The ephrin/Eph family executes various biological functions by regulating multiple downstream pathways [11]. For example, ephrinA1-induced activation of EphA2 stimulates the MAP/ERK kinase signaling cascade [46], EphB4 receptor signaling mediates endothelial cell migration and proliferation via the PI_3_K/Akt pathway [47], and the mTOR pathway is a crucial mediator of EphA signaling in neurons [48]. Because mTOR is known to be an important downstream molecule of Akt, we hypothesized that the Akt/mTOR pathway can be activated by ephrinA1/EphA1 and thus become involved in the homing process of EPCs to HCC. Using specific chemical pathway inhibitors to investigate the role of the Akt/mTOR pathway, we found that the blockage of the PI_3_K/Akt and mTOR pathways remarkably suppressed EphA1-activated SDF-1 expression and secretion, suggesting that the Akt/mTOR pathway mediates the up-regulation of SDF-1 by EphA1. Our results were in accordance with a previous study that observed the involvement of the ephrinA1/EphA1-activated PI3K/Akt pathway in chemokine-mediated T lymphocyte migration [49].

In summary, the most significant and novel finding of this study is that the activation of the ephrinA1/EphA1 pathway in HCC cells can significantly increase the expression and secretion of SDF-1 in the tumor microenvironment, thus promoting tumor angiogenesis by enhancing the homing and tube formation abilities of EPCs in a paracrine fashion. The homing of EPCs in tumor angiogenesis is a multi-step process involving the interactions and mutual influence of multiple factors and complicated signal transduction pathways. Our identification of the signal pathway and its function in HCC progression could be breakthrough that sheds new light on tumor anti-angiogenesis therapy.

## Conclusions

Our findings indicate that ephrinA1/EphA1 pathway activation in HCC cells can promote tumor angiogenesis by enhancing the homing and tube formation abilities of EPCs in a paracrine fashion, and targeting the EphA1/SDF-1 signaling pathway might be a therapeutic anti-angiogenesis approach for treating HCC.

## Methods

### Cell culture

After EPCs were isolated from peripheral blood, they were expanded ex vivo, as previously described [50]. Early passage EPCs (passage 2) were seeded in flasks pre-coated with type I rat tail collagen and cultured in complete EGM-2 medium (Lonza, NJ, USA). Human umbilical vein endothelial cells (HUVECs) were isolated from human umbilical venous blood via collagenase digestion and then cultured in complete EGM-2 medium in flasks coated with type I collagen. All cultures were maintained in a humidified incubator at 37 °C in an atmosphere containing 5 % CO_2_. Passage 2 EPCs and passage 2 HUVECs were used in the related experiments in this study. In addition, two human hepatoma cell lines, HLE (EphA1-negative) and Huh-7 (EphA1-positive) were obtained from the Japan Health Science Research Resources Bank (HSRRB, Osaka, Japan). HLE was cultured in complete MEM medium containing 10 % fetal bovine serum (FBS; Gibco, NY, USA). The Huh-7 was cultured in 10 % FBS-containing Dulbecco’s Modified Eagle’s Medium (DMEM).

### Animal studies

The Wenzhou Medical University Committee for the Use and Care of Animals approved all animal experiment procedures prior to the study. Forty-eight 6- to 7-week-old male C57BL/6 nude mice (Center for Laboratory Animals, Wenzhou Medical University, Wenzhou, China) were randomly divided into four groups for xenograft implantation at the right flank of the fourth inguinal mammary. The Group 1 mice were injected with IgG-Fc-treated HLE cells as controls. The Group 2 mice were injected subcutaneously with 1× 10^7^ HLE cells stimulated with 5 mg/ml EphA1-Fc, while the Group 3 and 4 mice were injected with the same amount of EphA1-Fc-stimulated HLE cells transfected with SDF-1 specific siRNA (GenPharma, Shanghai, China) or control scrRNA (GenPharma, Shanghai, China), respectively. Tumor development was monitored regularly by observing tumor growth and measuring the tumor size with a caliper once every 3 days. At post-xenograft Day 14, all of the mice were injected with 5 × 10^5^ EPCs through tail veins, at a time when endogenous tumor angiogenesis was completed [51]. Prior to injection, the EPCs were co-incubated with 2 g/mL DiI-ac-LDL (Biomedical Technologies, Stoughton, Mass) for 1 h. At post-xenograft Day 36, all of the mice were sacrificed for tumor removal. The tumor volume was determined using the formula volume = width^2^ × length × 0.52. A portion of the harvested tumor tissue was fixed in 10 % buffered formalin and embedded in paraffin for immunohistochemical (IHC) analysis, and the rest was snap-frozen in liquid nitrogen and stored at −80 °C.

### Preparation of conditioned medium (CM-HLE and CM-Huh-7)

Huh-7 cells transfected with EphA1 siRNA or EphA1 scrRNA were prepared as previously described [20]. To prepare the transfected HLE cells, after they had grown to 70 %-80 % confluency, the cells were washed three times with phosphate buffered saline (PBS) and then incubated in 0.5 % FBS-containing DMEM medium for 48 h. For the IgG-Fc (R&D, MN, USA) and ephrinA1-Fc (R&D, MN, USA) activated HLE cell groups, after the cells reached 50–60 % confluency, they were washed three times with PBS and then incubated in 5 mg/ml IgG-Fc or ephrinA1-Fc MEM medium containing 0.5 % FBS for 48 h. The cells were washed three more times with PBS and then cultured in 0.5 % FBS-containing MEM medium for another 48 h. For SDF-1 specific siRNA or scramble siRNA transfection, the ephrinA1-Fc activated HLE cells were incubated with SDF-1 specific siRNA or scrambled siRNA (scrRNA) for 6 h and then cultured for another 48 h after the medium was replaced with fresh MEM medium containing 0.5 % FBS. The conditioned media of both the transfected HLE and Huh-7 cell cultures were harvested and centrifuged at 1000 rpm for 10 min to remove cell debris. After being filtered through a 0.22-μm filter, the conditioned media were stored at 4 °C for subsequent use.

### Co-culture experiments

After stimulation with 5 mg/ml IgG-Fc or ephrinA1-Fc for 48 h, the EphA1-negative HLE cells were washed three times and cultured in 0.5 % FBS-containing medium. The EPCs were cultured on cell culture inserts with a 0.4-μm pore size (BD Biosciences, CA, USA) at a density of 1 × 10^5^ cells/insert in 0.5 % FBS-containing medium. After 24 h of equilibration, the inserts containing the EPCs were added to the plates, with the pretreated HLE cells in the lower compartment. The cells were co-cultured for 48 h to allow the diffusion of the medium components without cell migration. The EPCs were then collected for immunofluorescence (IF) and western blot (WB) assays. All of the independent experiments were conducted in triplicate.

### RNA extraction and quantitative real-time reverse transcription polymerase chain reaction (RT-PCR) analysis

Total RNA was extracted using the RNAeasy mini kit (Qiagen, CA, USA). cDNA synthesis was performed using the High-Capacity cDNA Reverse Transcription kit from Applied Biosystems (Grand Island, NY, USA) to transcribe 2 μg of total RNA. RT-PCR was used to semi-quantify the mRNA levels using an ABI 7300 system. The assay was performed with specific primers at 95 °C for 10 min, followed by 38 cycles of 95 °C for 15 s and 60 °C for 60 s. The mRNA level was determined before and after treatment with the comparative ΔΔCt method using 18S ribosomal RNA as the normalization gene. The statistical significance and standard error were determined using the ΔCt values. All reactions were performed in triplicate. The primer sequences are listed in Table 1.

### Western blot analysis

The siEphA1/Huh-7 cells and scrEphA1/Huh-7 cells were cultured overnight in DMEM medium containing 0.5 % FBS. The HLE cells were stimulated with 5 mg/ml EphrinA1-Fc or 5 mg/ml IgG-Fc in MEM medium containing 0.5 % FBS for 48 h, with the latter serving as a negative control. Subsequently, the ephrinA1-Fc-stimulated HLE cells were treated with 2 ng/ml LY294002 (SIGMA, MO, USA), a phosphoinositide 3-kinases (P1_3_K)/Akt inhibitor, or 2 ng/ml rapamycin (SIGMA, MO, USA), an mTOR signaling pathway inhibitor, for 24 h. The cells were then washed and lysed using lysis buffer. After protein quantification with a bicinchoninic acid (BCA) protein assay kit (Pierce, IL, USA), equal amounts of protein (40 μg/lane) were separated using 4–15 % sodium dodecyl sulfate-polyacrylamide gel electrophoresis (SDS-PAGE) and transferred onto polyvinylidene fluoride (PVDF) membranes (Millipore, MA, USA). After blocking for 1 h with 5 % BSA and washing with TBST, the membranes were probed with polyclonal or monoclonal antibodies against EphA1 (1:1000), EphA2 (1:1000), SDF-1 (1:1000), ERK (1:1000), phospho-ERK (1:1000), Akt (1:1000), phospho-Ake1:1000), mTOR(1:1000), phospho-mTOR (1:1000), or β-actin (1:5000) via incubation at 4°C overnight. Subsequently, goat anti-mouse or goat anti-rabbit horseradish peroxidase (HRP)-conjugated secondary antibodies (1:10000) were used to detect antigen antibody complexes. Immune complexes were visualized using the HyGLO HRP detection kit from Denville Scientific (Metuchen, NJ, USA) and exposed to X-ray films. β-actin was detected to verify equal loading. Each experiment was repeated at least three times. All antibodies are listed in Additional file 3: Table S1.

### SDF-1 and CXCR4 co-immunoprecipitation (IP)

EPCs collected from the co-culture experiment were washed twice with ice-cold PBS and lysed via incubation on ice for 30 min in 1 ml of modified RIPA lysis buffer containing 50 mM Tris–HCl, pH 7.4, 1 % NP-40, 0.25 % Na-deoxycholate, 150 mM NaCl, 1 mM EDTA, 1 mM PMSF, aprotinin, leupeptin, and pepstatin: 1 μg/ml each,1 mM Na_3_VO_4_, 1 mM NaF, and a commercial protease inhibitor mixture (Complete-Mini Protease Inhibitor Mixture; Roche Diagnostics). After the proteins were quantified and the concentration was adjusted with PBS to approximately 2 μg/μl, the cell lysate was pre-cleared by adding 20 μl of protein G sepharose bead slurry per ml of cell lysate and incubating at 4 °C for 1 h on a rocker. SDF-1 and CXCR4 proteins were immunoprecipitated by incubating pre-cleared lysate with rabbit anti-SDF-1 antibody or mouse anti-CXCR4 antibody and protein G-Sepharose (40 μl) overnight at 4 °C. The immune complexes were pelleted by centrifugation for 1 min at 14,000 × *g*, washed three times with lysis buffer, and released from the beads by boiling for 5 min in 40 μl 2× sample buffer. The beads were collected using centrifugation, and the supernatants were resolved with SDS-PAGE and subjected to WB analysis, as described in the preceding section.

### IHC staining

For IHC staining, the tumor tissues were fixed in formalin, embedded in paraffin, cut into 4-μm sections, and stained using antibodies against CD31 (1:600). Negative controls were established by substituting the primary antibody with 1 % BSA-TBS. To quantify the tumor microvascular density (MVD), CD31-positive cells were identified by a brown precipitate in the cytoplasm of the endothelial cells, and the vessels in each section were counted in five independent microscope fields [52].

### IF staining and confocal microscopy

Sections of HCC cells and tumor tissues were permeabilized with 0.1 % Triton X-100 in PBS for 30 min. The sections were then blocked with 5 % goat serum for 1 h at room temperature and incubated with anti-CD31 or anti-SDF-1 overnight at 4 °C. Next, the sections were rinsed in PBS and incubated for 1 h at room temperature with Alexa Fluor 488-conjugated anti-mouse and Alexa Fluor 594-conjugated anti-rabbit IgG from Molecular Probes (Grand Island, NY, USA) and Invitrogen (Carlsbad, CA, USA), respectively. The sections were mounted with Prolong Gold anti-fade reagent with DAPI from Invitrogen (Carlsbad, CA, USA).

The aforementioned EPCs were seeded on Falcon™ eight-well culture slides. After rinsing with Dulbecco’s PBS (D-PBS) at room temperature, the cells were fixed with 2.5 % formaldehyde in PIPES for 20 min. They were then rinsed with D-PBS and blocked with 5 % normal goat serum for 1 h at 37°C. After incubation with antibodies against SDF-1, CXCR4, EphA1, CD31, CD133, CD45, CD90, VEGFR2, or eNOS for 1 h at 37°C, the cells were processed IF procedures similar to those described above. The slides were examined under a confocal microscope (Zeiss LSM 510, Carl Zeiss Inc., Thornwood, NJ). For the negative control sections, the primary antibody was replaced with PBS.

### Enzyme-linked immunosorbent assay (ELISA)

The conditioned media were collected from the siEphA1/Huh-7, scrEphA1/Huh-7, and siEphA2/Huh-7, and scrEphA2/Huh-7 cell cultures and from the HLE cell culture in which the cells were incubated in low serum medium (0.5 % FBS) for 48 h after stimulation in low serum medium (0.5 % FBS) MEM medium containing 5 mg/ml IgG-Fc or ephrinA1-Fc for 48 h. The SDF-1 concentrations were measured using a human SDF-1 immunoassay kit from Abcam (Cambridge, MA, USA).

### Cell chemotaxis assay

The chemotaxis of EPCs to different HCC cell groups was measured using the Transwell assay according to the following procedures. First, 5 × 10^4^ EPCs in 500 μL of serum-free medium were seeded in a 24-well chamber with an 8-μm pore size (BD Biosciences, CA, USA). Subsequently, siEphA1 and scrEphA1-transfected Huh-7 cells and ephrinA1-Fc- or IgG-Fc- stimulated HLE cells were seeded in the lower compartment. After 24 h of culturing at 37 °C, the cells on the upper surface of membrane were completely wiped away with a cotton swab. The migrated cells adhering to the lower surface of the membrane were fixed with 100 % methanol, stained with crystal violet and counted under a light microscope. The migrated cells were counted in five randomly selected microscopic fields for each chamber, and chemotaxis was expressed as the number of migrated cells per field.

### EPCs incorporation assay

EPCs and HUVECs were used for tube formation assays as described previously [53]. EPCs were labeled with Dil-ac-LDL (Molecular Probes, 2 ug/mL) at 37 °C for 20 min. After washing with PBS, 1,000 of the Dil-ac-LDL labeled EPCs were mixed with 10,000 of HUVECs in 100 uL of 10 % FBS/EGM-2 MV medium (Lonza) in order to evaluate the contribution of EPCs to Endothelial Cells derived tube formation. 100uL of cell suspensions were applied to 50 μL of Matrigel coated Falcon™ eight-well culture slides and then incubated for 24 h. After this, cell were fixed, followed by mounting with Prolong Gold anti-fade DAPI. The slides were examined and counted under a confocal microscope (Zeiss LSM 510, Carl Zeiss Inc., Thornwood, NJ).

### Cell tube formation assay

First, growth factor-reduced Matrigel (BD Biosciences, San Jose, CA, USA) was thawed and placed in a precooled 96-well plate. The plate was then kept at 37 °C for 1 h to allow the matrix solution to gel. After culturing in conditioned medium from the 0.5 % FBS-containing siEphA1- or scrEphA1-transfected Huh-7 cell cultures and ephrinA1-Fc- or IgG-Fc-stimulated HLE cell cultures for 12 h, the EPCs were resuspended in the respective conditioned media at a density of 1 × 10^5^ cells/mL. Subsequently, 100 μL of the cell suspension was loaded onto the surface of the gelled matrix and incubated at 37 °C for 8 h. The tubule length was measured using Image J image analysis software downloaded from the NIH website (Bethesda, MD, USA). In another set of experiments, the EPCs were incubated with conditioned media from the HLE cell cultures that were stimulated with 5 mg/ml ephrinA1-Fc or IgG-Fc and then transfected with SDF-1-specific siRNA or control siRNA. Each experiment was performed in triplicate.

### Wound healing assay

Cell migration ability was tested using a wound healing assay. SiEphA1/Huh-7 cells, scrEphA1/Huh-7 cells and ephrinA1-Fc- or IgG-Fc-stimulated HLE cells in their corresponding 0.5 % FBS-containing medium were seeded at a density of 1 × 10^5^ cells/well in a six-well plate and incubated until an even monolayer at 90 % confluence was achieved. Two mechanical scratches were made to create uniformly sized wounds in all wells. Imaging of the healing wounds was performed at 0, 24 and 48 h post-scratch. The percentage of wound closure was calculated for each well, and the data were normalized to controls.

### Statistical analysis

The results are expressed as the means ± standard deviation (SD). Multiple comparisons were conducted using the Student *t*-test. Statistical significances were accepted at *p < 0.05 and **p < 0.001.

## Additional files

Additional file 1: Figure S1. Identification of EPCs. A. Representative images of EPCs at the 7th day (top panel) and 21st (bottom panel) day after separation from peripheral blood. B. EPCs were characterized according to the presence of endothelial-specific markers, such as CD31, CD133, eNOS, or VEGFR2, or the absence of mesenchymal-specific markers, such as CD45 and CD90. Scale bar: 50 μm. C. Representative images of EPC’s uptake of DiI-ac-LDL and binding of FITC-UEA-1. Scale bar: 50 μm. D. Representative images showing the capacity of EPCs to induce tube formation on Matrigel. (JPG 95 kb) Additional file 2: Figure S2. EphrinA1-Fc increases EphA1 expression in HLE cells. A. WB assay of EphA1 and EphA2 expressed in HLE cells after activation with IgG-Fc or ephrinA1-Fc, with β-actin expression as a control for protein loading. B. IF staining of EphA1 and EphA2 expressed in HLE cells after activation with IgG-Fc or ephrinA1-Fc. Scale bar: 10 μm. C. EphA2 knockdown in Huh-7 cells. A. EphA2 mRNA expression in Huh-7 cells after EphA2 knockdown by EphA2 siRNA, determined with RT-PCR. B. Bar graph shows EphA2 mRNA expression after EphA2 scrRNA (black bar) and EphA2 siRNA transfection (white bar). The data represent the mean ± SD of three independent experiments. *Asterisks* indicate significant differences (****P* < 0.001). D. Huh-7 cells with different EphA2 expression levels: The result is normalized to the expression in Huh-7 cells transfected with EphA2 scrRNA for each chemokine. (JPG 154 kb) Additional file 3: Table S1. List of antibodies used in the study. (DOCX 15 kb)

## References

[1] Ferlay JSI, Ervik M, Dikshit R, Eser S, Mathers C, Rebelo M, Parkin DM, Forman D, Bray F (2013). GLOBOCAN 2012 v1.0, Cancer Incidence and Mortality Worldwide: IARC CancerBase No. 11 [Internet].

[2] Folkman J (2007). Angiogenesis: an organizing principle for drug discovery?. Nat Rev Drug Discov.

[3] Llovet JM, Ricci S, Mazzaferro V, Hilgard P, Gane E, Blanc JF, de Oliveira AC, Santoro A, Raoul JL, Forner A, Schwartz M, Porta C, Zeuzem S, Bolondi L, Greten TF, Galle PR (2008). Sorafenib in advanced hepatocellular carcinoma. N Engl J Med.

[4] Liang PH, Tian F, Lu Y, Duan B, Stolz DB, Li LY (2011). Vascular endothelial growth inhibitor (VEGI; TNFSF15) inhibits bone marrow-derived endothelial progenitor cell incorporation into Lewis lung carcinoma tumors. Angiogenesis.

[5] Nguyen MP, Lee D, Lee SH, Lee HE, Lee HY, Lee YM (2015). Deguelin inhibits vasculogenic function of endothelial progenitor cells in tumor progression and metastasis via suppression of focal adhesion. Oncotarget.

[6] Suriano R, Chaudhuri D, Johnson RS, Lambers E, Ashok BT, Kishore R, Tiwari RK (2008). 17Beta-estradiol mobilizes bone marrow-derived endothelial progenitor cells to tumors. Cancer Res.

[7] Vajkoczy P, Blum S, Lamparter M, Mailhammer R, Erber R, Engelhardt B, Vestweber D, Hatzopoulos AK (2003). Multistep nature of microvascular recruitment of ex vivo-expanded embryonic endothelial progenitor cells during tumor angiogenesis. J Exp Med.

[8] Gao D, Nolan DJ, Mellick AS, Bambino K, McDonnell K, Mittal V (2008). Endothelial progenitor cells control the angiogenic switch in mouse lung metastasis. Science.

[9] Li CX, Shao Y, Ng KT, Liu XB, Ling CC, Ma YY, Geng W, Fan ST, Lo CM, Man K (2012). FTY720 suppresses liver tumor metastasis by reducing the population of circulating endothelial progenitor cells. PLoS One.

[10] Zhu Z, Chen G, Li X, Yin Q, Yang Z, Yi J (2012). Endothelial progenitor cells homing to the orthotopic implanted liver tumor of nude mice. Journal of Huazhong University of Science and Technology Medical sciences = Hua zhong ke ji da xue xue bao Yi xue Ying De wen ban = Huazhong keji daxue xuebao Yixue Yingdewen ban.

[11] Pasquale EB (2010). Eph receptors and ephrins in cancer: bidirectional signalling and beyond. Nat Rev Cancer.

[12] Zhang J, Hughes S (2006). Role of the ephrin and Eph receptor tyrosine kinase families in angiogenesis and development of the cardiovascular system. J Pathol.

[13] Foubert P, Silvestre JS, Souttou B, Barateau V, Martin C, Ebrahimian TG, Lere-Dean C, Contreres JO, Sulpice E, Levy BI, Plouet J, Tobelem G, Le Ricousse-Roussanne S (2007). PSGL-1-mediated activation of EphB4 increases the proangiogenic potential of endothelial progenitor cells. J Clin Invest.

[14] Giannoni E, Taddei ML, Parri M, Bianchini F, Santosuosso M, Grifantini R, Fibbi G, Mazzanti B, Calorini L, Chiarugi P (2013). EphA2-mediated mesenchymal–amoeboid transition induced by endothelial progenitor cells enhances metastatic spread due to cancer-associated fibroblasts. J Mol Med.

[15] Wang J, Dong Y, Wang X, Ma H, Sheng Z, Li G, Lu G, Sugimura H, Zhou X (2010). Expression of EphA1 in gastric carcinomas is associated with metastasis and survival. Oncol Rep.

[16] Iida H, Honda M, Kawai HF, Yamashita T, Shirota Y, Wang BC, Miao H, Kaneko S (2005). Ephrin-A1 expression contributes to the malignant characteristics of {alpha}-fetoprotein producing hepatocellular carcinoma. Gut.

[17] Fox BP, Tabone CJ, Kandpal RP (2006). Potential clinical relevance of Eph receptors and ephrin ligands expressed in prostate carcinoma cell lines. Biochem Biophys Res Commun.

[18] Wang J, Ma J, Dong Y, Shen Z, Ma H, Wang X, Shi S, Wu J, Lu G, Peng L (2013). High expression of EphA1 in esophageal squamous cell carcinoma is associated with lymph node metastasis and advanced disease. Apmis.

[19] Maru Y, Hirai H, Takaku F (1990). Overexpression confers an oncogenic potential upon the eph gene. Oncogene.

[20] Chen G, Wang Y, Zhou M, Shi H, Yu Z, Zhu Y, Yu F (2010). EphA1 receptor silencing by small interfering RNA has antiangiogenic and antitumor efficacy in hepatocellular carcinoma. Oncol Rep.

[21] Hristov M, Weber C (2004). Endothelial progenitor cells: characterization, pathophysiology, and possible clinical relevance. J Cell Mol Med.

[22] Appleby SL, Cockshell MP, Pippal JB, Thompson EJ, Barrett JM, Tooley K, Sen S, Sun WY, Grose R and Nicholson I. Characterization of a distinct population of circulating human non-adherent endothelial forming cells and their recruitment via intercellular adhesion molecule-3. PLoS One. 2012;7(11):e46996. 10.1371/journal.pone.0046996 PMC3492591 23144795

[23] Foubert P, Squiban C, Holler V, Buard V, Dean C, Levy BI, Benderitter M, Silvestre JS, Tobelem G, Tamarat R (2015). Strategies to Enhance the Efficiency of Endothelial Progenitor Cell Therapy by Ephrin B2 Pretreatment and Coadministration with Smooth Muscle Progenitor Cells on Vascular Function During the Wound-Healing Process in Irradiated or Nonirradiated Condition. Cell Transplant.

[24] Funk SD, Yurdagul A, Albert P, Traylor JG, Jin L, Chen J, Orr AW (2012). EphA2 activation promotes the endothelial cell inflammatory response: a potential role in atherosclerosis. Arterioscler Thromb Vasc Biol.

[25] Peplow PV (2014). Influence of growth factors and cytokines on angiogenic function of endothelial progenitor cells: a review of in vitro human studies. Growth factors (Chur, Switzerland).

[26] Shih Y-T, Wang M-C, Zhou J, Peng H-H, Lee D-Y, Chiu J-J. Endothelial progenitors promote hepatocarcinoma intrahepatic metastasis through monocyte chemotactic protein-1 induction of microRNA-21. Gut. 2015;64(7):1132-47. 10.1136/gutjnl-2013-306302 24939570

[27] Yang P, Li QJ, Feng Y, Zhang Y, Markowitz GJ, Ning S, Deng Y, Zhao J, Jiang S, Yuan Y, Wang HY, Cheng SQ, Xie D, Wang XF (2012). TGF-beta-miR-34a-CCL22 signaling-induced Treg cell recruitment promotes venous metastases of HBV-positive hepatocellular carcinoma. Cancer Cell.

[28] Ling CC, Ng KT, Shao Y, Geng W, Xiao JW, Liu H, Li CX, Liu XB, Ma YY, Yeung WH, Qi X, Yu J, Wong N, Zhai Y, Chan SC, Poon RT (2014). Post-transplant endothelial progenitor cell mobilization via CXCL10/CXCR3 signaling promotes liver tumor growth. J Hepatol.

[29] Monnier J, Boissan M, L’Helgoualc’h A, Lacombe M-L, Turlin B, Zucman-Rossi J, Théret N, Piquet-Pellorce C, Samson M (2012). CXCR7 is up-regulated in human and murine hepatocellular carcinoma and is specifically expressed by endothelial cells. Eur J Cancer.

[30] Chen Y, Huang Y, Reiberger T, Duyverman AM, Huang P, Samuel R, Hiddingh L, Roberge S, Koppel C, Lauwers GY, Zhu AX, Jain RK, Duda DG (2014). Differential effects of sorafenib on liver versus tumor fibrosis mediated by stromal-derived factor 1 alpha/C-X-C receptor type 4 axis and myeloid differentiation antigen-positive myeloid cell infiltration in mice. Hepatology.

[31] Schneider C, Teufel A, Yevsa T, Staib F, Hohmeyer A, Walenda G, Zimmermann HW, Vucur M, Huss S, Gassler N, Wasmuth HE, Lira SA, Zender L, Luedde T, Trautwein C, Tacke F (2012). Adaptive immunity suppresses formation and progression of diethylnitrosamine-induced liver cancer. Gut.

[32] Gao Q, Zhao Y-J, Wang X-Y, Qiu S-J, Shi Y-H, Sun J, Yi Y, Shi J-Y, Shi G-M, Ding Z-B (2012). CXCR6 upregulation contributes to a proinflammatory tumor microenvironment that drives metastasis and poor patient outcomes in hepatocellular carcinoma. Cancer Res.

[33] Tang KH, Ma S, Lee TK, Chan YP, Kwan PS, Tong CM, Ng IO, Man K, To KF, Lai PB (2012). CD133+ liver tumor-initiating cells promote tumor angiogenesis, growth, and self-renewal through neurotensin/interleukin-8/CXCL1 signaling. Hepatology.

[34] Zhou SL, Dai Z, Zhou ZJ, Wang XY, Yang GH, Wang Z, Huang XW, Fan J, Zhou J (2012). Overexpression of CXCL5 mediates neutrophil infiltration and indicates poor prognosis for hepatocellular carcinoma. Hepatology.

[35] Chen K-J, Lin S-Z, Zhou L, Xie H-Y, Zhou W-H, Taki-Eldin A, Zheng S-S (2011). Selective recruitment of regulatory T cell through CCR6-CCL20 in hepatocellular carcinoma fosters tumor progression and predicts poor prognosis. PLoS One.

[36] Cui XD, Lee MJ, Yu GR, Kim IH, Yu HC, Song EY, Kim DG (2010). EFNA1 ligand and its receptor EphA2: potential biomarkers for hepatocellular carcinoma. International journal of cancer Journal international du cancer.

[37] Melero-Martin JM, Dudley AC (2011). Concise review: Vascular stem cells and tumor angiogenesis. Stem Cells.

[38] Ping YF, Bian XW (2011). Consice review: Contribution of cancer stem cells to neovascularization. Stem Cells.

[39] Salvucci O, de la Luz SM, Martina JA, McCormick PJ, Tosato G (2006). EphB2 and EphB4 receptors forward signaling promotes SDF-1-induced endothelial cell chemotaxis and branching remodeling. Blood.

[40] Yamaguchi J-i, Kusano KF, Masuo O, Kawamoto A, Silver M, Murasawa S, Bosch-Marce M, Masuda H, Losordo DW, Isner JM (2003). Stromal cell–derived factor-1 effects on ex vivo expanded endothelial progenitor cell recruitment for ischemic neovascularization. Circulation.

[41] Song T, Dou C, Jia Y, Tu K, Zheng X (2015). TIMP-1 activated carcinoma-associated fibroblasts inhibit tumor apoptosis by activating SDF1/CXCR4 signaling in hepatocellular carcinoma. Oncotarget.

[42] Hsu WH, Chen CN, Huang HI, Lai YL, Teng CY, Kuo WH (2012). Urokinase induces stromal cell-derived factor-1 expression in human hepatocellular carcinoma cells. J Cell Physiol.

[43] Zhu H, Sun Q, Tan C, Xu M, Dai Z, Wang Z, Fan J, Zhou J (2014). Tacrolimus promotes hepatocellular carcinoma and enhances CXCR4/SDF-1α expression in vivo. Molecular medicine reports.

[44] Isozaki T, Arbab AS, Haas CS, Amin MA, Arendt MD, Koch AE, Ruth JH (2013). Evidence that CXCL16 is a potent mediator of angiogenesis and is involved in endothelial progenitor cell chemotaxis: studies in mice with K/BxN serum–induced arthritis. Arthritis & Rheumatism.

[45] Ishida Y, Kimura A, Kuninaka Y, Inui M, Matsushima K, Mukaida N, Kondo T (2012). Pivotal role of the CCL5/CCR5 interaction for recruitment of endothelial progenitor cells in mouse wound healing. J Clin Invest.

[46] Pratt RL, Kinch MS (2002). Activation of the EphA2 tyrosine kinase stimulates the MAP/ERK kinase signaling cascade. Oncogene.

[47] Steinle JJ, Meininger CJ, Forough R, Wu G, Wu MH, Granger HJ (2002). Eph B4 receptor signaling mediates endothelial cell migration and proliferation via the phosphatidylinositol 3-kinase pathway. J Biol Chem.

[48] Sahin M (2010). Eph receptor and mTOR pathway crosstalk: implications for cancer. Cell Cycle.

[49] Hjorthaug HS, Aasheim HC (2007). Ephrin-A1 stimulates migration of CD8 + CCR7+ T lymphocytes. Eur J Immunol.

[50] Griese DP, Ehsan A, Melo LG, Kong D, Zhang L, Mann MJ, Pratt RE, Mulligan RC, Dzau VJ (2003). Isolation and transplantation of autologous circulating endothelial cells into denuded vessels and prosthetic grafts implications for cell-based vascular therapy. Circulation.

[51] Folkman J (2002). Role of angiogenesis in tumor growth and metastasis. Semin Oncol.

[52] Weidner N, Semple JP, Welch WR, Folkman J (1991). Tumor angiogenesis and metastasis--correlation in invasive breast carcinoma. N Engl J Med.

[53] Yang J, Ii M, Kamei N, Alev C, Kwon SM, Kawamoto A, Akimaru H, Masuda H, Sawa Y, Asahara T (2011). CD34+ cells represent highly functional endothelial progenitor cells in murine bone marrow. PLoS One.

