# Supplementary figures and images for: EphA1 activation promotes the homing of endothelial progenitor cells to hepatocellular carcinoma for tumor neovascularization through the SDF-1/CXCR4 signaling pathway
Source: J Exp Clin Cancer Res. 2016 Apr 11;35:65. doi: 10.1186/s13046-016-0339-6 (PMC4827226; doi:10.1186/s13046-016-0339-6)

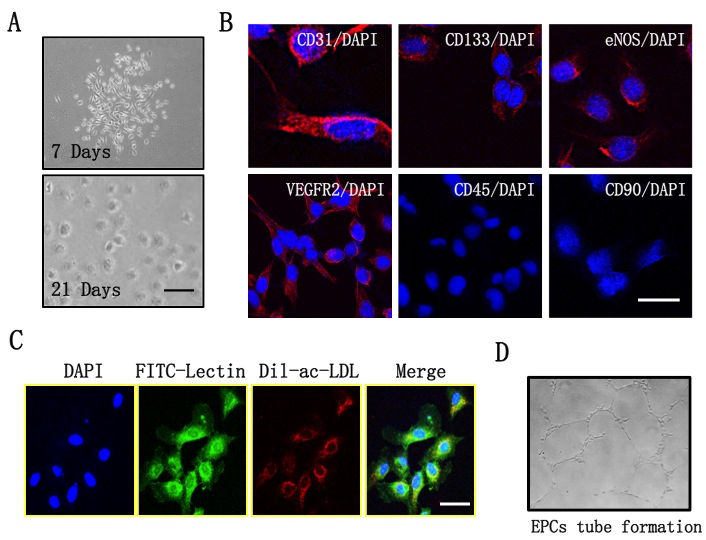

Supplement: Additional file 1: Figure S1. — Identification of EPCs. A. Representative images of EPCs at the 7th day (top panel) and 21st (bottom panel) day after separation from peripheral blood. B. EPCs were characterized according to the presence of endothelial-specific markers, such as CD31, CD133, eNOS, or VEGFR2, or the absence of mesenchymal-specific markers, such as CD45 and CD90. Scale bar: 50 μm. C. Representative images of EPC’s uptake of DiI-ac-LDL and binding of FITC-UEA-1. Scale bar: 50 μm. D. Representative images showing the capacity of EPCs to induce tube formation on Matrigel. (JPG 95 kb) [file 13046_2016_339_MOESM1_ESM.jpg]

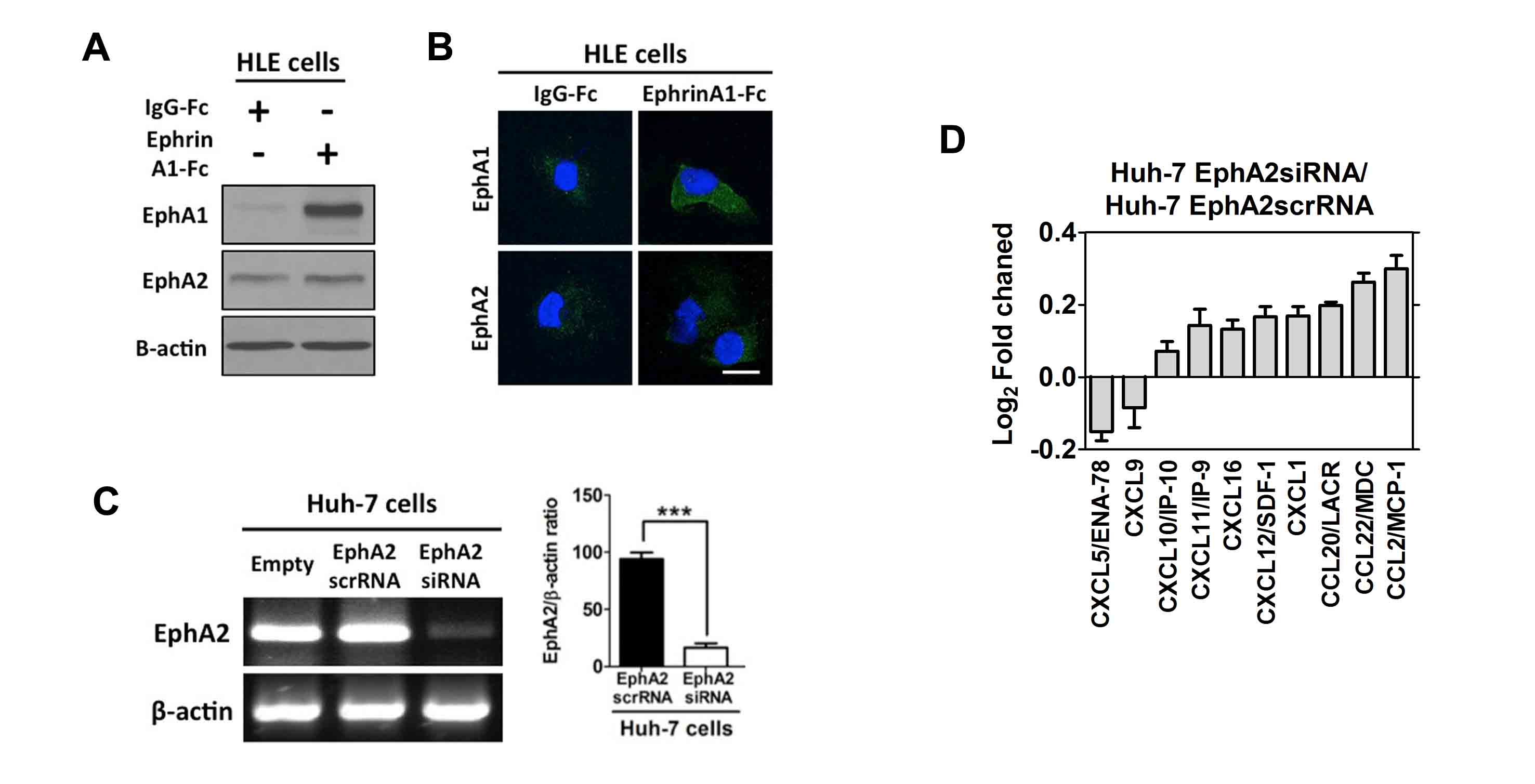

Supplement: Additional file 2: Figure S2. — EphrinA1-Fc increases EphA1 expression in HLE cells. A. WB assay of EphA1 and EphA2 expressed in HLE cells after activation with IgG-Fc or ephrinA1-Fc, with β-actin expression as a control for protein loading. B. IF staining of EphA1 and EphA2 expressed in HLE cells after activation with IgG-Fc or ephrinA1-Fc. Scale bar: 10 μm. C. EphA2 knockdown in Huh-7 cells. A. EphA2 mRNA expression in Huh-7 cells after EphA2 knockdown by EphA2 siRNA, determined with RT-PCR. B. Bar graph shows EphA2 mRNA expression after EphA2 scrRNA (black bar) and EphA2 siRNA transfection (white bar). The data represent the mean ± SD of three independent experiments. Asterisks indicate significant differences (***P < 0.001). D. Huh-7 cells with different EphA2 expression levels: The result is normalized to the expression in Huh-7 cells transfected with EphA2 scrRNA for each chemokine. (JPG 154 kb) [file 13046_2016_339_MOESM2_ESM.jpg]
