# Supplementary material for: EphA1 activation promotes the homing of endothelial progenitor cells to hepatocellular carcinoma for tumor neovascularization through the SDF-1/CXCR4 signaling pathway
Source: J Exp Clin Cancer Res. 2016 Apr 11;35:65. doi: 10.1186/s13046-016-0339-6 (PMC4827226; doi:10.1186/s13046-016-0339-6)
Supplement: Additional file 3: Table S1. — List of antibodies used in the study. (DOCX 15 kb) [file 13046_2016_339_MOESM3_ESM.docx]

**Additional file 3: Table S1 List of antibodies used in the study**

| **ANTIBODIES** | | | |
| --- | --- | --- | --- |
| **Antibodies** | **Dilution** | **Source** | **Address** |
| EphA1 | 1:1000 WB 1:200 IF | AB65074, ABCAM | Cambridge, MA, USA |
| EphA2 | 1:1000 WB 1:200 IF | AB150304, ABCAM | Cambridge, MA, USA |
| ERK | 1:1000 WB, 1:200 IHC 1:100 IF | 9101, Cell Signaling | Danvers, MA, USA |
| Phosphor ERK | 1:1000 WB, 1:100 IF | 5013, Cell Signaling | Danvers, MA, USA |
| Akt | 1:1000 WB | 9572, Cell Signaling | Danvers, MA, USA |
| Phosphor Akt S473 | 1:1000 WB | 4060, Cell Signaling | Danvers, MA, USA |
| mTOR | 1:1000 WB | 2972, Cell Signaling | Danvers, MA, USA |
| Phosphor mTOR S2448 | 1:1000 WB | 2971, Cell Signaling | Danvers, MA, USA |
| SDF-1/CXCL12 | 1:1000 WB 1:200 IF | AB9797, ABCAM | Cambridge, MA, USA |
| CXCR4 | 1:1000 WB 1:200 IF | AB10403, ABCAM | Cambridge, MA, USA |
| CD 31 | 1:100 IHC 1:200 IF | AB187376, ABCAM | Cambridge, MA, USA |
| CD133 | 1:200 IF | AB66141, ABCAM | Cambridge, MA, USA |
| CD45 | 1:200 IF | AB195722, ABCAM | Cambridge, MA, USA |
| CD90 | 1:200 IF | AB33700, ABCAM | Cambridge, MA, USA |
| VEGFR2 | 1:200 IF | 9698, Cell Signaling | Danvers, MA, USA |
| eNOS | 1:200 IF | AB66127, ABCAM | Cambridge, MA, USA |
| Beta Actin | 1: 5000 WB | A5441, Sigma | St. Louis, MO, USA |
